# Comparative Study of Nanostructured Multilayer Cr/(Cr/a-C)ml Coatings Deposited on HS6-5-2 Steel by Magnetron Sputtering

**DOI:** 10.3390/ma19061073

**Published:** 2026-03-11

**Authors:** Rayna Dimitrova, Krum Petrov, Yavor Sofronov, Valentin Mishev, Milko Angelov, Boriana Tzaneva, Boyan Dochev, Antonio Nikolov, Milko Yordanov, Krassimir Marchev

**Affiliations:** 1 Department of Material Science and Technology, Faculty of Industrial Technology, Technical University of Sofia, 1756 Sofia, Bulgaria; kpetrov@tu-sofia.bg (K.P.); v_mishev@tu-sofia.bg (V.M.); anikolov@tu-sofia.bg (A.N.); 2 Department of Theory of Mechanisms and Machines, Faculty of Industrial Technology, Technical University of Sofia, 1756 Sofia, Bulgaria; 3 Center of Excellence “Mechatronics and Clean Technology”—Campus Studentski Grad, Technical University of Sofia, 1756 Sofia, Bulgaria; borianatz@tu-sofia.bg; 4 Faculty of Industrial Technology, Technical University of Sofia, 1756 Sofia, Bulgaria; milko.angelov@tu-sofia.bg (M.A.); k.marchev@northeastern.edu (K.M.); 5 Department of Chemistry, Faculty of Electronic Engineering and Technologies, Technical University of Sofia, 1756 Sofia, Bulgaria; 6 Department of Mechanics, Faculty of Mechanical Engineering, Technical University of Sofia—Branch Plovdiv, 4000 Plovdiv, Bulgaria; dochev@tu-plovdiv.bg; 7 Department of Mechanical Engineering, Manufacturing Engineering and Thermal Engineering, Faculty of Engineering and Pedagogy—Branch Sliven, Technical University of Sofia, 8800 Sliven, Bulgaria; m_yordanov@tu-sofia.bg; 8 College of Professional Studies, Northeastern University, Boston, MA 02115, USA

**Keywords:** nanostructured multilayer coatings, Cr/(Cr/a-C)ml coatings, mechanical properties, magnetron sputtering deposition, HS6-5-2 steel

## Abstract

Comparative analysis of nanostructured multilayer Cr/(Cr/a-C)ml coatings on HS6-5-2 steel was carried out. The coatings were deposited at various chromium target power values using PVD technology, particularly the magnetron sputtering method. The effect of different technological regimes on the properties of the nanostructured multilayer Cr/(Cr/a-C)ml coatings was studied. Identical characterization methods were used for the three types of coatings obtained. Cross-sections of the coated samples were prepared in order to directly determine the thickness of the resulting coatings, their uniformity, and the presence of defects or imperfections, both at the substrate–coating interface and within the coatings themselves. Calotest and Daimler-Benz adhesion test were also performed to evaluate the coated layers’ thickness and evaluate their adhesion strength. Scanning electron microscopy (SEM) and energy-dispersive X-ray spectroscopy (EDX) analyses were carried out to define the chemical composition of the multilayered coatings. To evaluate the hardness and modulus of elasticity of the resulting coatings, nanoindentation measurements were also conducted. The data obtained under the three different deposition regimes were analyzed and compared, which allowed us to assess the influence of the chromium target power during the deposition process on the properties of the obtained coatings.

## 1. Introduction

It is generally accepted that the term nanostructured material refers to a metallographic structure with crystals/grains that have a nanoscale size (<100 nm) in at least one of their three-dimensional directions. Unlike typical metal alloys with micro/macrograin structures, ultrafine-grain (UFG) and nanostructured materials have unique physical, chemical, and mechanical properties, which make them suitable for a wide range of applications [1]. The nanoscale structure may be characteristic of the entire volume of the material, but it is particularly applicable to the formation of only nanoscale thin films on the surface of the substrate using various chemical, physical, and metallurgical methods. In most cases, the substrate/base also has enhanced mechanical properties—superalloys for the aviation industry [1], tool steels for manufacturing processes [2], etc.—which work under high temperatures combined with high loads. In addition, nanoscale coatings provide extra resistance against mechanical wear, thermal stresses, and mechanical or fatigue microcracks formation [1,3].

According to their composition, nanostructured coatings are metal-based coatings (nanocrystalline metals—Ni, Cu, Ti, Al, Cr; alloys—Ni-P, Ni-Co, Ti-Al, Cr-Al) which provide high hardness, wear resistance, and improved corrosion resistance; ceramic coatings (oxides—Al_2_O_3_, TiO_2_, ZrO_2_, SiO_2_; carbides—TiC, SiC, WC; nitrides—TiN, CrN, AlN), which are known for their thermal stability, chemical resistance, and high hardness; and composite coatings (metal–ceramic composites—Ni-Al_2_O_3_, Ni-SiC, Ti-AlN; polymer–ceramic composites), which combine the toughness of metals/polymers with the hardness of ceramics [1,4,5]. The composition of coatings is of great importance for their properties (mechanical performance, chemical stability, optical, electrical properties, etc.) and for their specific application.

According to their architecture, they can be divided into single layers (monolithic, composite, gradient), with reduced efficiency due to their low adhesion to the substrate, and multilayer (microscale, nanoscale, superlattice) [2,6]. Multilayer coatings are systems consisting of successive layers of two or more materials forming a periodic structure. Nanocomposites consist of nanocrystals of transition metal nitrides or carbides that are dispersed randomly in an amorphous matrix, with the two materials being insoluble in each other. An example is nc-TiN:a-Si3N4, nc-TiC:a-C (nc-TiC:a-C:H). Nanolaminates (superlattices) consist of alternatively arranged layers of two materials with a large difference in shear modulus, with a layer thickness of several nanometers and a sharp interface between the layers [7]. A multilayer material where the thickness of the individual layers is comparable to the lattice constant is called a superlattice (SL) [8]. The creation of nanoscale coatings through layer-by-layer assembly (each with a thickness of several nm) is particularly effective, as is the combination of different methods of deposition for the individual layers. They contain several functional layers with different mechanical behaviors due to a better stress distribution and greater fracture toughness, since the energy of the crack is dissipated by reflection and branching when it propagates through the boundaries between layers [2,9]. It is known that multilayer films with nanometric periods improve the mechanical properties of thin film coatings by enhancing their hardness or toughness and by relaxing the stress in the film [10,11,12].

According to their behavior in relation to their distance from the substrate, the layers may vary in thickness and have different strictly defined functions. For metal forming/cutting tools, they can be defined as metallic adhesives for creating a metallurgical bond (e.g., Ni, Ti, Cr, Mo, Zr); a base layer with high hardness and low residual stresses (e.g., TiN, CrN, TiCN, ZrN); a thermally insulating layer (e.g., TiAlN, TiZrN); and a surface layer with a low coefficient of friction and high wear resistance (e.g., CrCN, AlTiVN, TiN, CrNi) [2,9,13,14].

Carbon-based coatings can be classified as graphite-like (mainly sp2 bonding) and diamond-like—DLC (large proportion of sp3 bonding). Carbon coatings have high hardness, chemical inertness, and excellent tribological properties [14,15,16,17,18]. The main problem is related to the low adhesion to the crystal lattice of tool steels and the resulting high surface stresses at the coating/substrate interface, which are due to the large differences in their hardness and Young’s modulus. To keep the strain in the coating and the substrate as close as possible to each other, almost identical values of the modulus of elasticity are required for both, so that the internal stress in the system is minimized and the substrate can deform elastically together with the coating, without cracks or delamination occurring. Achieving a higher H (hardness)-to-E (modulus of elasticity) ratio also reduces the possibility of residual stress formation at the boundary between the coating and the substrate, which is related to improved adhesion [14,19,20,21,22].

Multilayer coating structure is an applicable approach to avoid low adhesion by combining the different properties of each individual layer [23,24], and obtaining a nanocomposite multilayer structure is related to the alloying of the hard ceramic layers [25] or their alternation with more plastic metal layers (Cr, Ta, Ti) [26,27].

Chromium-based coatings have an excellent wear resistance, sufficient strength, and good adhesion to the substrate [28]. The chromium content usually provides very good corrosion resistance due to the formation of a passive layer of chromium oxide. Chromium carbide coatings have a low coefficient of friction and a higher H/E ratio [27,29,30,31]. Attempts have been made to determine whether wear is related to the H/E ratio of hard coatings, as predicting the H/E ratio for hard PVD coatings may demonstrate their ability to withstand substrate stress, which is indicative of their wear-resistant properties [21,32]. It is also supposed that the hardness-to-modulus of elasticity ratio (H/E), which is inversely proportional to the plasticity index, can be used to assess the wear resistance of materials. Considering that material removal is the result of plastic deformation during wear, a material with a high H^3^/E^2^ ratio, which indicates good resistance to plastic deformation (resistance to plastic flow), has an excellent wear resistance [32,33].

Chromium (Cr) is a carbide-forming element that interacts actively with carbon [34], and chromium carbides have structures representing combinations of three different stable phases—one cubic Cr_23_C_6_ and two orthorhombic Cr_7_C_3_ and Cr_3_C_2_ (with best mechanical properties)—and sometimes other metastable cubic CrC phases with a face-centered cubic lattice [11]. Some studies indicate that the deposition temperature is a decisive parameter in the synthesis of these phases, as their crystallization requires a temperature of at least 500 °C [9,10,35]. The excellent mechanical properties are retained even at high temperatures; therefore, chromium carbide and amorphous non-hydrogenated carbon Cr/(Cr/a-C) coatings are also used as surface layers in multilayer coatings. There are multilayer carbon composite films based on other metals, such as W/a-C, Mo/a-C, Ti/a-C, Al/a-C], Cu/a-C, a-CN/a-C, a-C/a-C, WCx/a-C, TiCx/a-C, TiNx/a-C и TiB2/a-C, which also have excellent properties compared to monolithic a-C films [23].

Depending on the application and the required property complex, various approaches are used to improve the properties of steel. These include surface modification methods, such as Plasma Nitriding; the synthesis of nanostructured coatings by the Chemical Vapor Deposition Method; the Physical Vapor Deposition Method; thermal evaporation; cathodic arc deposition; magnetron sputtering; and Pulsed Laser Deposition [1,36].

Physical Vapor Deposition (PVD) technology offers a number of advantages, such as low deposition temperatures, compatibility with various substrates, and the ability to produce smooth, dense coatings with precise control over the coating thickness, as well as the composition and stress. Magnetron sputtering, as a part of advanced PVD technologies, is one of the most widely used methods due to its excellent control over the coating properties [21,37].

The present paper considers nanostructured multilayer Cr/(Cr/a-C) ml coatings obtained by PVD technology. The coatings were deposited on high-speed steel (HS6-5-2) using a magnetron sputtering process at three different chromium target power regimes.

High-speed steels are used to produce cutting tools that operate under significant mechanical loading and at high cutting speeds. High-speed steels are characterized by a unique combination of elevated-temperature hardness, secondary hardening, a complex carbide structure, high wear resistance, good impact toughness, excellent hardenability, and microstructural stability under cyclic thermal loading. The main distinguishing feature of these steels is their high heat resistance (600–700 °C) combined with high hardness (above 60 HRC) and wear resistance, which is achieved through specific alloying and complex heat treatment. Tungsten and molybdenum are the main alloying elements that provide heat resistance in high-speed steels. Molybdenum has a comparable effect to that of tungsten and can partially replace it (in a ratio of Mo:W = 1:1.5), since tungsten is a relatively scarce and expensive alloying element. It is considered that the best properties are achieved in tools in which the alloying condition of Σ(W + 1.5 Mo) = 12–13% is fulfilled. Based on this principle, the HS6-5-2 steel was developed [38].

The aim of this work is to compare the data obtained for the three resulting coatings deposited on the same material substrate—HS6-5-2 steel—using medium-frequency (MF) pulsed DC power supplies operating at a fixed frequency, and to assess the effect of the variable chromium target power values on the chromium content, thickness, structure, and mechanical properties of the resulting nanostructured multilayer Cr/(Cr/a-C) ml coatings.

## 2. Materials and Methods

### 2.1. Materials

Tungsten–molybdenum high-speed tool steel HS6-5-2 (HSS, 1.3343, BDS EN ISO 4957:2018 [39]) samples with dimensions of 24 mm × 24 mm × 3 mm were used as substrates for nanostructured multilayer Cr/(Cr/a-C)ml coating deposition by the magnetron sputtering process. Three samples were prepared for each coating deposition regime: R1, R2 and R3 (9 samples in total).

High-speed HS6-5-2 steel is one of the most widely used HSS grades due to its excellent properties. This steel is characterized by exceptional cutting performance and thermal stability and can be categorized as tool steel with a high content of alloying elements—a precisely balanced system of strong carbide-forming elements—W (5.9–6.7 wt.%), Mo (4.7–5.2 wt.%), Cr (3.8–4.5 wt. %), and V (1.7–2.1 wt. %) [40,41,42,43].

Tungsten and molybdenum form M_6_C carbide, which partially dissolves in the γ-solid solution during austenitization and which after hardening contributes to the formation of alloyed martensite. Together with vanadium, they prevent martensite decomposition during heating and ensure the necessary heat resistance. Undissolved M_6_C carbides increase the wear resistance of steel. Vanadium forms extremely hard VC (MeC) carbide during tempering, which partially dissolves in austenite and increases the heat resistance and hardness of the steel as a result of the precipitation hardening effect. The undissolved part of MeC carbide increases the wear resistance of the steel. Chromium forms M_23_C_6_ carbide, which dissolves completely at the heating temperature during quenching. Its main role is to improve the hardenability of high-speed steels. Chromium also affects the carbide formation processes during tempering [38,44].

The specific features outlined above clearly justify the choice of HS6-5-2 as a substrate material for deposition of nanostructured multilayer Cr/(Cr/a-C)ml coatings by the magnetron sputtering process. The significant hardness at elevated temperatures, stable structure, and carbide strengthening of HS6-5-2 provide the necessary load-bearing capacity and adhesion for Cr/(Cr/a-C) ml coatings, enhancing crack and delamination resistance in multilayer architectures [38,40], [45,46,47,48].

The chemical composition of HS6-5-2 steel used as a substrate was determined using the Oxford Instruments FOUNDRY-MASTER UV apparatus (Abingdon/High Wycombe, Oxfordshire, UK) and is presented in Table 1.

**Figure 1:**
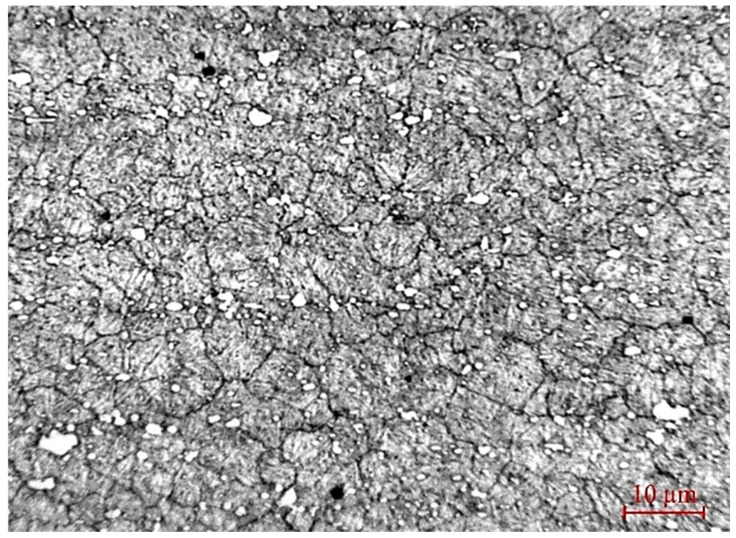
Microstructure of HS6-5-2 steel substrate.

The microstructure of the HS6-5-2 steel base shown in Figure 1 was revealed using a 3% solution of nitric acid in ethanol and observed using the optical microscope Neophot 21 (Carl Zeiss Jena, Jena, Germany).

After etching with a 3% solution of nitric acid in ethanol, the martensitic matrix appears dark, retained austenite is visible as lighter regions, and carbides are observed as bright particles due to their higher resistance to the etchant. The carbides seen in the metallographic image vary in size. The coarser carbides represent primary carbides, precipitated during crystallization of the melt. The finer ones, which are predominant, are secondary carbides, precipitated during tempering after quenching. Finer carbides reduce the risk of crack formation and propagation compared to coarse carbides, which can be potential stress concentrators. The fine dispersion of small carbides suggests improved wear resistance and toughness of the steel. Nevertheless, even small carbides can cause problems if they cluster or segregate at grain boundaries. There is also a low density of non-metallic inclusions in the metal matrix, which suggests high-quality refining of the steel. Non-metallic inclusions can be distinguished morphologically. They are observed as dark gray-to-black particles against the metallic matrix; however, their chemical composition cannot be determined by optical microscopy. A large number of non-metallic inclusions, as well as their grouping, provides conditions for the formation of cracks, reducing fatigue strength and toughness.

### 2.2. Preparation of Sample Surfaces and Coating Deposition

The substrate surfaces were pretreated, followed by coating deposition, with both operations being performed in accordance with the procedures presented in previous research [49].

Nanostructured multilayer Cr/(Cr/a-C)ml coatings were deposited to the substrates using a magnetron sputtering system developed at the Center of Excellence “Mechatronics and Clean Technologies” at the Technical University of Sofia [50].

The coating deposition process uses three operating regimes of the magnetron system with a single variable—the value of the power supplied to the chromium target in the final stage of coating deposition (R1—135 W; R2—175 W; and R3—500 W).

The choice of Cr target power is based on previous studies [51] for the same coating on stainless steels, where the composition is specified in atomic percents. The objective is to achieve a Cr/C ratio of ≈ 50/50 at.%. The selected Cr target power values were chosen to control the Cr/C ratio in the multilayer structure. Due to the nonlinear dependence of the Cr/C ratio on the applied target powers, caused by differences in sputtering yields and mutual redeposition effects, the desired composition cannot be predicted by a linear model.

The novelty of the present study is related to the use of MF pulsed DC power supplies operating at a fixed frequency of 100 kHz and a specific pulse shape, similar to HiPIMS pulses’ shape for the deposition of nanostructured multilayer Cr/(Cr/a-C)ml coatings. Various approaches for depositing such coatings have been reported in the literature, with many studies using direct current (DC) or low-frequency pulsed mode, often in combination with reactive gas atmospheres [16,17,18,20,23,24,26,27,33,35]. In the present work, a non-reactive sputtering process under controlled pulsed conditions is applied, enabling reproducible control of the coating composition and structure. This aspect is particularly relevant with respect to process stability and potential scale-up for industrial applications.

### 2.3. Methods for Coating Characterization

The comparative characterization of the obtained nanostructured multilayer Cr/(Cr/a-C)ml coatings on HS-6-5-2 steel substrates by magnetron sputtering was performed through a number of experimental tests.

The chemical composition of the coatings, the coating thicknesses, adhesion to the substrate, and the mechanical properties of hardness and modulus of elasticity were determined.

To assess the uniformity of the coatings, obtained cross-sections of the coated samples were also prepared. The preparation includes several stages: cutting; preliminary electrochemical nickel plating of the cut specimens to prevent coating delamination during further processing; hot mounting; grinding; polishing; and etching. The cross-sections of the coated samples were observed via scanning electron microscopy (SEM) “Carl Zeiss” EVO MA 10 (Germany).

The chemical composition of deposited nanostructured multilayer Cr/(Cr/a-C)ml coatings was determined by scanning electron microscopy using SEM “Carl Zeiss” EVO MA 10 (Germany), as well as using energy-dispersive X-ray spectroscopy “Bruker” (Billerica, MA, USA).

The coating thicknesses were determined according to EN ISO 26423:2016 “Determination of coating thickness by crater-grinding method” [52], using the “KaloMAX II” calotester from BAQ GmbH (Berlin, Germany). The abrasive agent consists of 0.1 µm particle size diamond paste suspended in ethanol at a ratio of 1:4 (BAQ, Germany).

Adhesion of the coatings was determined according to EN ISO 26443:2024 “Rockwell indentation test for evaluation of adhesion of ceramic coatings” [53]. When the hardness of the substrate exceeded 54 HRC (62 HRC in this case), the test was performed according to scale “C” (diamond cone 120° diamond cone, 200 nm tip radius and load 150 kgf/1471 N) on a VEB WPM Leipzig (Leipzig, Saxony, Germany) hardness tester. The coating thicknesses and the indentation marks used for adhesion evaluation were examined using a Best Scope BS-6022TRF optical microscope (Shenzhen, China) equipped with Capture 2.1 software. Test reports were prepared according to the requirements of EN ISO 26423:2016 for coating thickness evaluation and EN ISO 26443:2024 for coating adhesion testing.

Mechanical properties of the studied coatings—hardness (H) and modulus of elasticity (E)—were determined by nanoindentation. Nanoindentation tests were performed using an Agilent Nano Indenter G200 (Agilent, Santa Clara, CA, USA) equipped with diamond Berkovich indenter. For each coating, a single nanoindentation test was performed at 12 uniformly distributed locations on the coating surface, resulting in 12 individual measurements. Each indentation was performed under the following test parameters: loading/unloading test mode, Berkovich indenter, test load of 5 mN, number of load/unload cycles one, loading speed of 0.5 mN/s, load hold time at maximum force of 5 s, and unloading speed of 0.5 mN/s. The elasticity index (H/E ratio), resistance to plastic deformation index (H^3^/E^2^ ratio) and resistance to the crack formation index (1/(HE^2^) ratio) related to the tribological behavior of hard coatings [21,32] were also determined.

## 3. Results and Discussion

**Figure 2:**
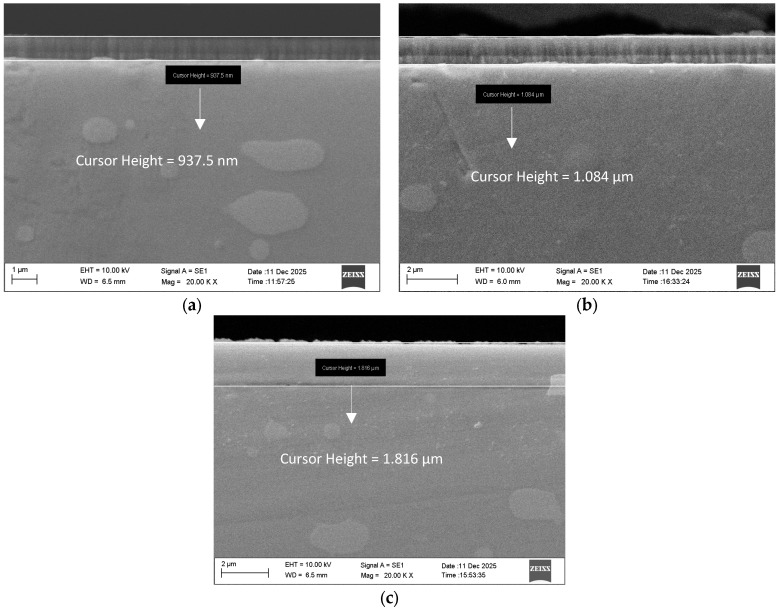
Cross-sections of the examined coatings for R1 (**a**), R2 (**b**) and R3 (**c**) deposition regime.

The cross-sections of the examined Cr/(Cr/a-C)ml coatings are presented in Figure 2.

It can be seen that the obtained coatings have a thickness of 937.5 nm for the first (R1), 1.084 µm for the second (R2), and 1.816 µm for the third (R3) deposition regime. The deposited coatings are uniform and homogeneous and follow the profile of the substrate surface. No pores, cracks, interlayer micro-debonding, or voids are observed in the examined coatings.

**Figure 3:**
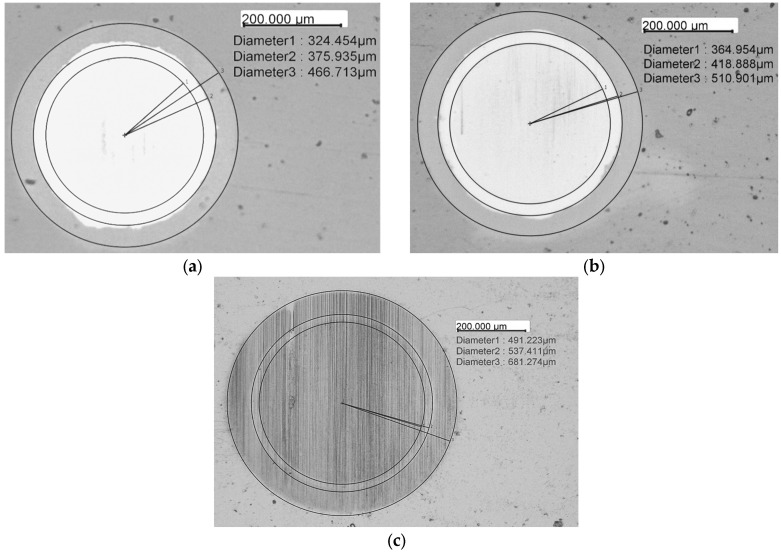
Determination of the individual layer thicknesses of the examined coatings after the Calotest for R1 (**a**), R2 (**b**) and R3 (**c**) deposition regimes according to EN ISO 26423:2016.

The results of determining the coating thickness are shown in Figure 3.

Five measurements were performed for each sample, and the results were averaged and compared with the SEM measurements and are presented in Table 2.

The results obtained in both measurements of coating thickness (SEM and Calotest) are almost identical. The increasing thickness of the adhesive Cr sublayer (R2 and R3) can be explained by the slower decrease in the power of the chromium target during the transition to the multilayer mode. On the other hand, the increase in the total thickness of the (Cr/a-C)_ml coating is related to the higher power of the chromium target during the deposition of the multilayer structure. A significant increase (nearly twice as high) in coating thickness is observed for the third deposition regime, R3, at 500 W power of the chromium target. The bilayer period was determined (Λ_1_ = 1.894 ± 0.261 nm for R1, Λ_2_ = 2.089 ± 0.250 nm for R2 and Λ_3_ = 3.903 ± 0.153 nm for R3, respectively) from the measured thickness of the multilayer coating and the deposition parameters (180 min process duration, 2 rpm substrate holder rotation speed), assuming that one bilayer is formed during each full rotation of the substrate holder.

**Figure 4:**
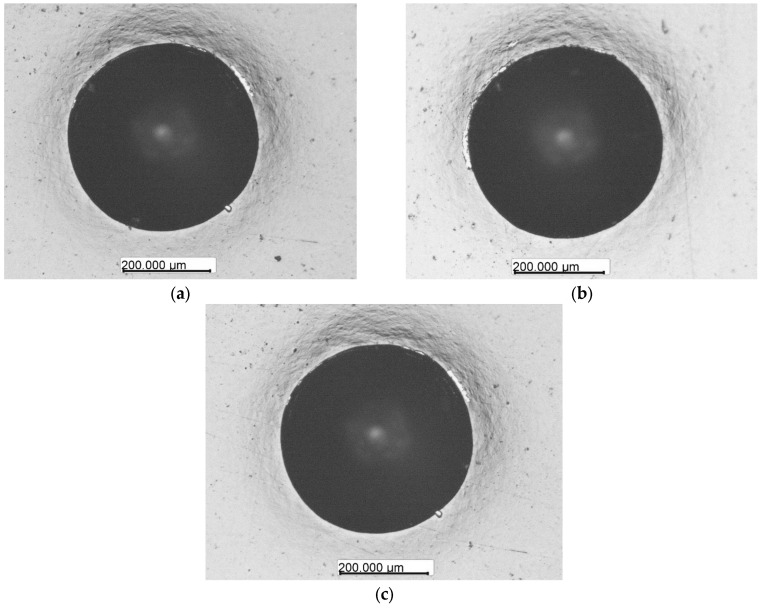
Determination of coating adhesion for R1 (**a**), R2 (**b**) and R3 (**c**) deposition regimes according to EN ISO 26443:2024.

The results of coating adhesion evaluation are presented in Figure 4.

Five measurements were performed for each sample. No radial cracks, spallation, or delamination from the coatings around the crater are observed in any of the three deposition regimes. Therefore, according to the EN ISO 26443:2024 criteria, all three coatings correspond to class 1—visible local plastic deformation around the indentation, but without cracks or delamination, which indicates maintained adhesion of the coating to the substrate.

**Figure 5:**
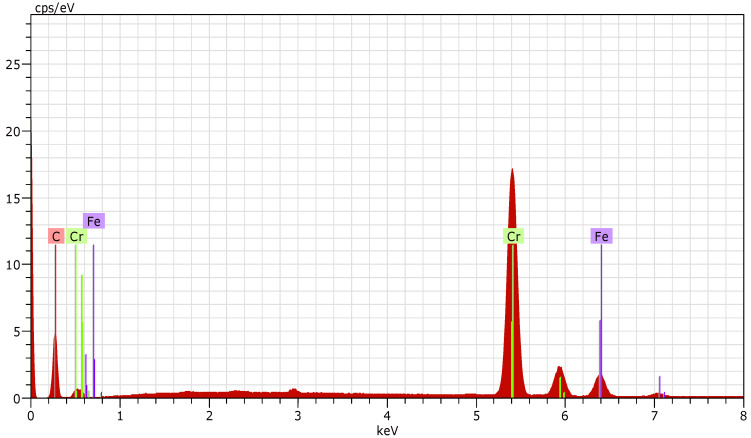
EDX of nanostructured multilayer Cr/(Cr/a-C)ml coating deposited on HS6-5-2—R1.

**Figure 6:**
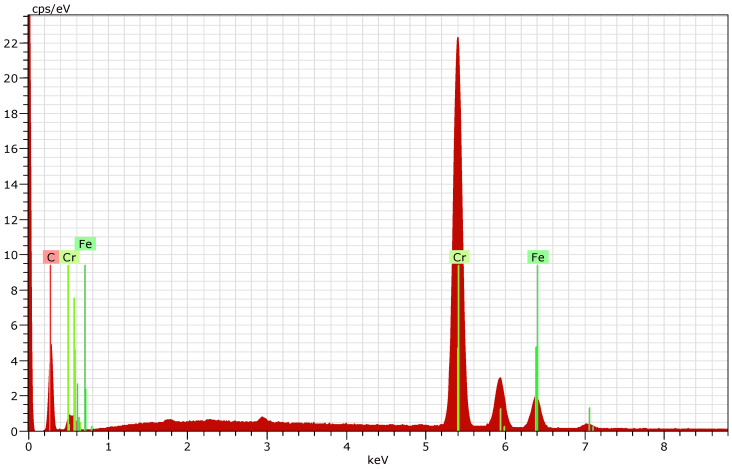
EDX of nanostructured multilayer Cr/(Cr/a-C)ml coating deposited on HS6-5-2—R2.

**Figure 7:**
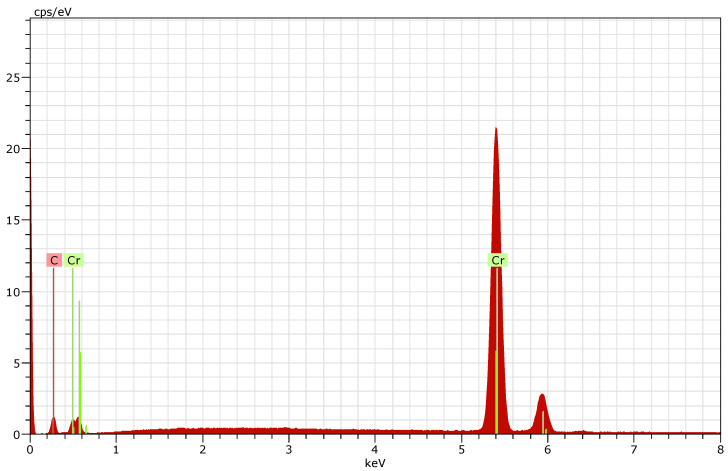
EDX of nanostructured multilayer Cr/(Cr/a-C)ml coating deposited on HS6-5-2—R3.

The results of the EDX analysis for each deposition regime (R1, R2, and R3) are presented in Figure 5, Figure 6 and Figure 7.

The presence of iron is detected at the first two operation regimes (R1 and R2), which is most likely due to the relatively low thickness of the deposited coatings.

In order to determine the elemental composition of the coated surfaces, five measurements were carried out for each sample. The elemental composition data obtained were averaged and are presented in Table 3.

By eliminating the presence of iron in the layers (obviously influenced by the substrate), the obtained values for the elemental composition for R1 and R2 are presented in Table 4. Compositional recalculation was required only for these two regimes, while no recalculation was necessary for R3 due to the absence of detectable iron.

The average values of hardness and modulus of elasticity obtained from the 12 measurements for each sample, as well as the calculated values of mechanical indices, are presented in Table 5.

Nanohardness testing by applying the indentation directly to the surface requires a specific ratio between the penetration depth and the thickness of the tested layer. This requires 10% or less penetration into the surface layer, thereby avoiding the influence of the substrate. This requirement for eliminating the influence of the substrate is particularly important in the case of ultra-thin films, even when they are much harder than the substrate. In some cases, when the thickness is similar, but the hardness of the coating is different, the penetration under identical test conditions, i.e., under the same load, may vary; accordingly, the requirement for a 10% ratio has not been met in some of the samples, as in the samples from R1 and R2. In such cases, in order to use the measurements correctly, it is necessary to apply an additional analytical calculation of the hardness of the coating itself. It is assumed that the measured hardness values are the so-called composite hardness, H_C_, which is a mathematical combination of the coating hardness, H_F_, and the substrate hardness, H_S_. Several dependencies have been proposed for determining the hardness in the composite coating/substrate system, which depends on the areas of plastic and elastic deformation. For thin coatings, this dependency has the following form [54,55]:

H_C_ = a · H_F_ + b · H_S_ (1)

under the fulfilled condition of a + b = 1, where a and b are coefficients that determine linear dependence.

Although nanohardness is generally higher than microhardness, due to the so-called indentation size effect [56], there are several variations in the proposed dependence for evaluating true hardness with different functions, including: Young’s modulus, microstructure, concentration gradient difference, or residual stresses [57]. Three of these dependencies are used to assess true hardness: the Jönsson and Hogmark model [58], the modified model of Korsunsky and the modified Puchi-Cabrera model. The geometric model of Jönsson and Hogmark is expressed by the quadratic dependence [58].

H_F_ = H_S_ + (H_C_ − H_F_)/(2C · t/h_C_ − C^2^(t/h_C_)^2^ (2)

where t—film thickness; H_F_—film hardness; H_S_—substrate hardness; H_C_—composite hardness; h_C—_indentation depth; and for very hard and brittle films, C = sin^2^11°.

This model is elegant and successful, but due to the indentation size effect, it is only applicable for microhardness testing [59]. In this case, however, we have nanoindentation with the same load of 5 mN, at which, for a chromium target power of 500 W (R3), there is a test with about 6–7% penetration of the layer thickness, which can be used to verify the model. In other cases, with lower power (R1 and R2), due to the lower thickness and lower hardness of the coating, the depth is between 14 and 17%, but the model can be verified, and the *C* coefficient can be refined.

**Figure 8:**
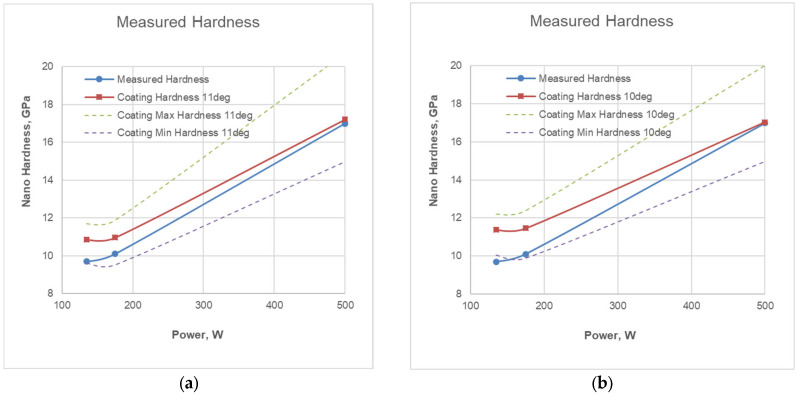
Determination of coating hardness H according to the Jönsson/Hogmark model with coefficient *C =* sin^2^11° (**a**) and *C =* sin^2^10° (**b**).

Figure 8 presents two variants of the Jönsson–Hogmark model for changing the hardness of the coating with different values of the coefficient *C*. In addition to the average values calculated according to (2), the maximum and minimum hardness values determined according to (2) have been plotted on the graph. The measured nanoindentation hardness values for the different coating deposition regimes are also shown for comparison.

As can be seen from Figure 8a, the values for R3 at a chromium target power of 500 W with a penetration of 6–7% of the layer thickness differ by about 0.2 GPa, while for the other two regimes, R1 and R2, the differences are 0.9 and 1.2 GPa, respectively. This suggests that the Jönsson/Hogmark model can also be used in nanoindentation, despite the so-called indentation size effect. To account for the effect of the difference in micro- and nanoindenter penetration and the coincidence of hardness values at R3, it is proposed to use a lower coefficient corresponding to *C =* sin^2^10*°*—see Figure 8b. Moreover, the differences with the other two regimes, R1 and R2, increase to 1.4 and 1.7 GPa, respectively, which is not inconsistent with the expected trend.

The modulus of elasticity determined by nanoindentation also depends on the penetration depth, and when it exceeds 10% of the coating thickness, the measured values begin to reflect the properties of the substrate. In such cases, the determined reduced modulus *E_r_*, calculated using the Oliver–Pharr method, cannot be determined according to the dependence (3) [60,61]:

(3) $$E_{r}=\frac{\sqrt{\pi }}{2\beta }\frac{S}{\sqrt{A_{c}}},$$

where *S*—stiffness; *A_C_*—projected contact area of the indenter; and *β*—coefficient accounting for the type of indenter.

The values of the modulus of elasticity for such penetration, *E_r_*(*h*), vary depending on the ratio of the penetration depth *h_c_* to the coating thickness *t*, with “deep” penetration—the calculated modulus is that of the substrate, rather than the coating. The description of the variation in the modulus is performed using an exponential function of the ratio of penetration depth to coating thickness (Doerner–Nix exponential model) [62,63] of the following form:

(4) $$\frac{1}{E_{r}\left(h\right)}=\frac{1}{E_{r,s}}+\left(\frac{1}{E_{r,f}}-\frac{1}{E_{r,s}}\right)exp\left(-\alpha \frac{h_{c}}{t}\right),$$

where *E_r_*(*h*)—measured reduced modulus at penetration depth *h_c_*; *E_r,f_*—reduced modulus of the coating; *E_r,s_*—reduced modulus of the substrate; and *t*—thickness of the coating.

The reduced modulus of the coating, *E_r,f_*, is calculated by transforming expression (4).

The modulus of elasticity of the coating, *E_f_*, depends on Poisson’s ratios for the indenter, and the coating and is derived according to the dependence (5) [60,64].

(5) $$E_{f}=\frac{1-\nu_{f}^{2}}{\frac{1}{E_{r,f}}-\frac{1-\nu_{i}^{2}}{E_{i}}}$$

where *E_i_*—modulus of the indenter (assumed to be 1140 GPa); *ν_i_*—Poisson’s ratio for the indenter (assumed to be 0.07); and *ν_f_*—Poisson’s ratio for the coating (assumed to be 0.2).

**Figure 9:**
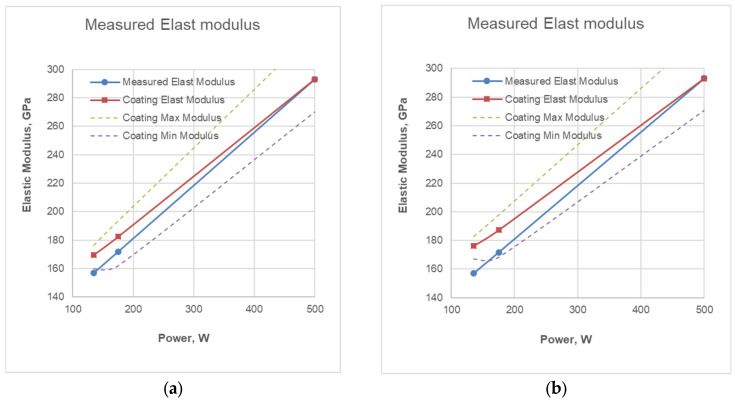
Determination of coating modulus of elasticity E according to the Doerner–Nix model with coefficients *β* = 1.024, *α =* 1.0, *ν_f_* = 0.2 (**a**) and *β* = 1.016, *α =* 1.5, *ν_f_* = 0.2 (**b**).

Figure 9 shows two configurations of the Doerner–Nix model for the variation in the modulus of elasticity at the typical *ν_f_* for ceramic coatings, but with different coefficients α and β. For clarity, the extreme calculated values of the coating elasticity modulus have been added, and for comparison, the values measured by nanoindentation at different coating deposition regimes are provided.

As can be seen from the figures, the reported differences between the measured and calculated values of the modulus of elasticity are in the following range: for regime R1—13 to 19 GPa; and for regime R2—11 to 16 GPa. The differences are determined by the penetration depth–coating thickness ratio, as well as by the values of the two coefficients used in the calculations.

**Figure 10:**
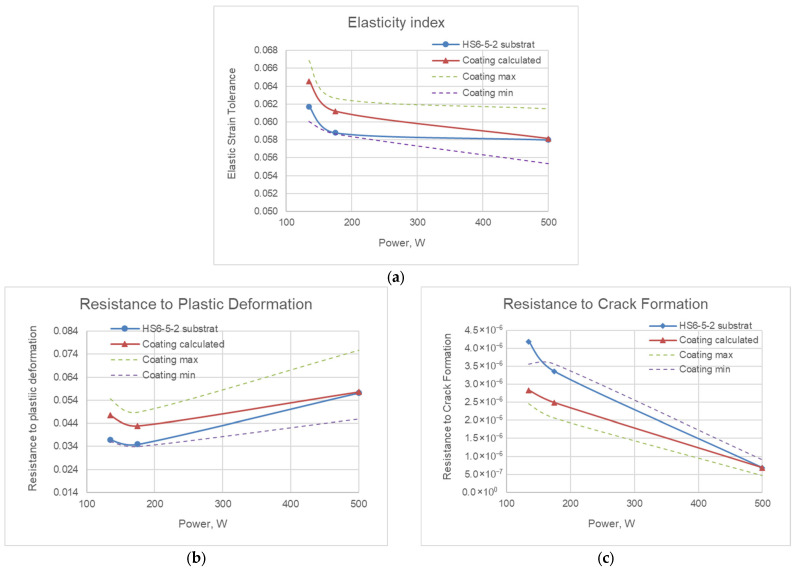
Determination of mechanical indices of deposited coatings: elasticity index (**a**), resistance to plastic deformation (**b**) and resistance to crack formation (**c**).

The results of the determination of mechanical indices—the elasticity index (H/E ratio), the resistance to plastic deformation index (H^3^/E^2^ ratio) and the resistance to crack formation index (1/(H·E^2^) ratio)—of the coatings for R1, R2 and R3 are presented in Figure 10.

The calculated average values of the Cr/(Cr/a-C)ml coating hardness, modulus of elasticity and the mechanical indices recalculated values (H/E, H^3^/E^2^ and 1/(H·E^2^)) are presented in Table 6.

The low hardness obtained in the first two deposition regimes, R1 and R2 (11.4–11.5 GPa), can be explained by the lack of clearly formed and sharply defined elementary layers in the multilayer structure.

Taking into account that the interatomic distances for both chromium and carbon are approximately 0.4 nm, and considering the recalculated data for the elemental composition of the layers (Table 4), as well as the values obtained for the bilayer periods, at R1 the thickness of a single layer of chromium is in the order of 0.642 nm, which is less than two atomic monolayers, and the thickness of a single layer of carbon is 1.252 nm, which is about three atomic monolayers. For R2, the thickness of a single layer of chromium is approximately 0.812 nm—about two atomic monolayers. The thickness of a single layer of carbon is 1.277 nm—also about three atomic monolayers. Due to the low thicknesses of the individual chromium and carbon layers and the constant roughness value of the individual layers (deposited under the same conditions), it can be assumed that under these deposition conditions, the requirement for a sharply defined interface between the individual layers is not met, and the deposited multilayer coatings cannot be considered as a superlattice, regardless of their thickness in the range of nanometers.

In the third deposition regime, R3, the thickness of a single layer of chromium is in the order of 2.728 nm, which is approximately seven atomic monolayers, while the thickness of a single layer of carbon is 1.175 nm, which is again about three atomic monolayers. In this case, the individual thicknesses of the chromium and carbon layers are large enough to form clear boundaries, but the requirement for approximately equal thickness of the individual layers is not met. This may explain the relatively low measured/calculated hardness of 17 GPa.

In a classic superlattice, the atomic content of chromium and carbon is approximately 50% for both elements, which is not a fact for either of the coatings. Based on measurements of the coating composition as a function of the chromium target power, it can be assumed that setting the chromium target power to 240–270 W will result in a coating with a chromium/carbon ratio of 50/50%. With approximately equal thicknesses of the elementary layers of chromium and carbon of three monolayers of atoms (about 1.2 nm) and a bilayer period Λ = 2.4–2.5 nm, an increase in the hardness of the layer to about 25.0–26.0 GPa can be expected, as can a Young’s modulus around 370–390 GPa.

Regarding the mechanical indices, the H/E values range from 0.058 to 0.065, which covers moderate resistance to contact loads and wear [32]. At higher values of the H^3^/E^2^ ratio, under the same contact loads, the material exhibits predominantly elastic behavior, and the H^3^/E^2^ parameter can be used as a quantitative measure of the surface’s resistance to plastic deformation and its load-bearing capacity. The decrease in the values of the 1/(H·E^2^) ratio is related to an increase in the crack formation resistance, as well as higher resistance to coating failure [21]. Comparing the values obtained for H^3^/E^2^ and 1/(H·E^2^) ratios for the three deposited coatings, it can be concluded that the coating deposited at R3 demonstrates the best performance with respect to resistance to plastic deformation and crack formation.

## 4. Conclusions

- The coatings deposited in the R3 process demonstrate the best mechanical properties compared to the other two processes (R1 and R2) in terms of their hardness, modulus of elasticity and mechanical indices: resistance to plastic deformation and resistance to crack formation.
- The relatively low hardness values of the obtained Cr/(Cr/a-C)ml coatings following the R1 and R2 processes are due to a combination of several interrelated factors. The main reason is the very low thickness of the chromium layer (less than or around two multilayers), which does not allow for the formation of continuous and clearly defined sublayers. As a result, the multilayer structure cannot be considered a real superlattice; therefore, the hardening mechanism is not realized.
- Although the thickness of the sublayers is sufficient to form clearly defined boundaries in the R3 process, they are disproportionate—the chromium layer is almost two and a half times thicker than the carbon layer, meaning that the requirement for approximately equal thicknesses of the sublayers within a single bilayer period is not met. This also contributes to reduced hardness.
- An additional influence on the measured hardness and Young’s modulus is caused by the lower intensity of ion bombardment in the first two regimes compared to the third, due to the lower power of the chromium target, as well as the much lower sputtering rate and degree of ionization of carbon compared to chromium. This leads to low bias current density and insufficient ion bombardment, which also contributes to low hardness values.
- The geometric model of Jönsson and Hogmark can be used to calculate the hardness of the coating when depositing a thin hard Cr/(Cr/a-C)ml layer on an HS6-5-2 steel substrate, to account for the indentation size effect in nanoindentation, with deviated values of the rule for up to 10% of the coating thickness penetration depth.

## Figures and Tables

**Table 1:** Chemical composition of the HS6-5-2 steel substrate (average values and standard deviations).

Element	Fe	C	Mn	Si	P	S	Ni	Cr	Cu	Mo	V	W	Co
Avg. (wt.%)	81.96	0.90	0.26	0.35	0.02	0.01	0.22	3.96	0.10	4.19	1.79	5.66	0.57
SD (%)	0.14	1.71	0.73	2.35	0.29	1.33	0.63	0.15	0.37	0.42	0.27	1.49	1.38

**Table 2:** Comparison of (Cr/a-C)ml PVD coating thickness measurement results (µm).

Deposition Regime	Cr Sublayer Thickness	(Cr/a-C)ml Coating Thickness	PVD Coating Total Thickness	SEM Measurements Total Thickness
R1	0.302 ± 0.009	0.682 ± 0.094	0.985 ± 0.094	0.938
R2	0.325 ± 0.047	0.752 ± 0.090	1.077 ± 0.102	1.084
R3	0.349 ± 0.048	1.405 ± 0.055	1.754 ± 0.086	1.816

**Table 3:** Elemental composition of nanostructured multilayer (Cr/a-C)ml coating deposited on HS6-5-2 steel substrate.

Element	Avg. at% R1	Avg. at% R2	Avg. at% R3
Cr	32.43	37.12	69.89
C	63.28	58.34	30.11
Fe	4.29	4.54	---
Total at%	100	100	100

**Table 4:** Recalculation of elemental composition of nanostructured multilayer (Cr/a-C)ml coating deposited on HS6-5-2 steel substrate.

Element	Avg. at% R1	Avg. at% R2
Cr	33.88	38.89
C	66.12	61.11
Fe	0.0	0.0
Total at%	100	100

**Table 5:** Measured hardness, modulus of elasticity and calculated mechanical indices (H/E, H^3^/E^2^ and 1/(H·E^2^)) of the Cr/(Cr/a-C)ml coatings deposited under three different regimes.

Regime	Displacement (nm)	H (GPa) Avg.	E (GPa) Avg.	H/E	H^3^/E^2^	1/(H·E^2^)
R1	160.3	9.7	157.0	0.062	0.0370	4.19 × 10^−6^
R2	156.1	10.1	171.8	0.059	0.0349	3.35 × 10^−6^
R3	116.4	17.0	293.1	0.058	0.0572	6.85 × 10^−7^

**Table 6:** Calculated hardness, modulus of elasticity and mechanical indices (H/E, H^3^/E^2^ and 1/(H·E^2^)) of the Cr/(Cr/a-C)ml coatings deposited under three different regimes.

Regime	H (GPa) Avg.	E (GPa) Avg.	H/E	H^3^/E^2^	1/(H·E^2^)
R1	11.4	176.2	0.065	0.047	2.83 × 10^−6^
R2	11.5	187.3	0.061	0.043	2.49 × 10^−6^
R3	17.0	293.0	0.058	0.057	6.83 × 10^−7^

## Data Availability

The original contributions presented in this study are included in the article. Further inquiries can be directed to the corresponding authors.

## Abbreviations

(Cr/a-C)ml	Chromium/amorphous carbon multilayer
PVD	Physical vapor deposition
SEM	Scanning electron microscopy
EDX	Energy-dispersive X-ray spectroscopy
UFG	Ultrafine grain
SL	Superlattice
DLC	Diamond-like carbon
H	Hardness
E	Modulus of elasticity (Young’s modulus)
HRC	Rockwell hardness, C scale
MF	Medium frequency
DC	Direct current
HSS	High speed steel
HiPIMS	High-Power Impulse Magnetron Sputtering
Λ	Bilayer period
H_C_	Composite hardness
H_F_	Coating hardness
H_S_	Substrate hardness
HV	Vickers hardness
